# Investigating the growth-promoting effects of entophytic fungi isolated from *Camellia luteflora* on tomato seedlings

**DOI:** 10.3389/fmicb.2025.1641696

**Published:** 2025-11-14

**Authors:** Xiaojie Bai, Pingping Yan, Chunxia Guo, Hang Yi, Bangyou Liu, Li Wang

**Affiliations:** 1 Administration of Chishui Alsophila National Nature Reserve, Chishui, China; 2 College of Life Sciences, Sichuan University, Chengdu, China; 3 Sanya Aviation and Tourism College, Sanya, China

**Keywords:** *Camellia luteflora*, entophytic fungi, entophytic fungal inocula, tomato seedlings, growth promoting effect

## Abstract

*Camellia luteflora*, an endangered plant species native to China, faces significant conservation challenges due to its limited habitat and vulnerability to pathogens. Investigating the entophytic fungal communities within *C. luteflora*, particularly those that promote plant growth and stress resilience, is crucial. Through systematic evaluation, the growth-promoting ability of entophytic fungi from *C. luteflora* and the effects of inoculation on the growth of tomato seedlings were investigated. Among the 35 isolated entophytic fungi, 7 strains exhibited the ability to synthesize indole-3-acetic acid (IAA), 24 strains showed nitrogen-fixing abilities, 27 strains were capable of producing protease, and 15 entophytic strains had a siderophore utilization (SU) value exceeding 10.00%. Furthermore, five strains were identified as capable of potassium solubilization, and seven strains as phosphorus solubilizers. Three strains demonstrated a dual ability for phosphorus and potassium solubilization. The entophytic fungal inocula derived from entophytic fungi CG-II-1, CJ-V-1, and DJ-I-4 resulted in significant enhancement of chlorophyll content in tomato seedlings, as well as promoting root development and biomass accumulation. This study found that CJ-V-1 (*Colletotrichum siamense*) and CG-II-1 (*Helotiales* sp.) have significant potential for promoting plant growth, creating opportunities for utilizing these strains as innovative resources for entophytic fungal inocula development.

## 1 Introduction

With the rapid development of modern agriculture, there is a burgeoning demand for environmentally benign and sustainable agricultural technologies (Lin and Chen, 2019). The excessive use of chemical fertilizers leads to soil pollution, a decline in microbial diversity, and food safety issues (Vandana et al., 2021). Recently, an urgent necessity has arisen for natural agricultural strategies to supplant the long-standing application of chemical fertilizers, with the aim of augmenting crop productivity and curtailing the environmental pollution engendered by these compounds (Nongbet et al., 2022).

Plant growth-promoting microorganisms (PGPMs) refer to microorganisms that can colonize inside plants and effectively promote growth, as well as enhance stress resistance (such as drought resistance and salt tolerance) and improve plant quality through various direct and indirect pathways (Dixit et al., 2022; Kashyap and Manzar, 2025). Its eco-friendly characteristics offer new ideas for alleviating problems such as pollution and soil degradation in traditional agriculture. In general, PGPMs can be bifurcated into two principal groups: plant growth-promoting (rhizo)bacteria (PGPRs or PGPBs) and plant growth-promoting fungi (PGPFs) (Kloepper and Schroth, 1978; Liu et al., 2024). Among them, plant entophytic fungi, as the core group of PGPFs, colonize healthy plant tissues at a certain stage or throughout their entire life cycle and are widely distributed across various plant species (Wilson, 1995). The mutualistic symbiotic relationship established between entophytic fungi and host plants is crucial for plant growth and development as well as ecosystem stability, making it an increasingly important focus of research in biological and ecological sciences (Manzar et al., 2024).

Numerous studies have demonstrated that entophytic fungi are capable of promoting plant growth and development through a plethora of mechanisms (Rodriguez et al., 2009; Jia et al., 2016). First, entophytic fungi can significantly promote plant biomass accumulation, root development, and disease resistance by secreting plant hormones such as indole-3-acetic acid (IAA). The entophytic fungus *Aspergillus flavus* CHS1 isolated from the roots of *Quinoa* sp. not only secretes indoleacetic acid (IAA) and ferredoxin, but also exhibits significant cross-species growth promotion effects in rice (Lubna et al., 2018). Shah et al. (2022) demonstrated that 23 entophytic fungal strains isolated from the roots of *Dendrobium longicornu* are capable of producing IAA, confirming their potential to promote the growth of both host and cross-genera plants by generating metabolites essential for plant growth. Secondly, entophytic fungi can also activate soil nutrients by secreting organic acids and enzymes, significantly improving the mineral nutrient efficiency of plants. The organic acids/enzymes they secrete can convert insoluble elements such as phosphorus and iron into absorbable forms (Mullen et al., 1998; Zheng et al., 2022). Zhang et al. (2018) screened three strains (PN8, PN12, and PN15) of entophytic bacteria with phosphorus solubilizing ability, which exerted a growth-promoting effect on the medicinal plant *Panax notoginseng*. Furthermore, entophytic fungi can aid plants in withstanding adverse stress by modulating hormone levels and producing antioxidants within plants. Under drought stress, entophytic fungi can increase the soluble sugar and proline content of wheat, reduce malondialdehyde levels, and thereby improve water absorption capacity (Malinowski and Belesky, 2000); under salt stress, they significantly increase the aboveground biomass and plant height of rice and barley (Baltruschat et al., 2008; Bu et al., 2012).

Endophytic fungi, due to their significant plant growth-promoting, soil-improving, and stress-regulatory functions, have become a core resource for the development of entophytic fungal inocula. Research has shown that entophytic fungal inocula prepared from *Aspergillus fumigatiaffinis* can increase the leaf area, plant height, and stem circumference of tobacco (*Nicotiana tabacum*) by 63.0, 57.0%, and 44.0%, respectively (Li et al., 2022), while entophytic fungal inocula derived from *Scytalidium* sp. significantly increased the biomass and survival rate of *Dendrobium officinale* (Zhao et al., 2012). The significant effects demonstrated by entophytic fungi through the above-mentioned multiple growth-promoting mechanisms fully confirm their key regulatory role in plant growth and development, which also gives entophytic fungus-derived entophytic fungal inocula broad application prospects in the medical and agrochemical fields (Saini et al., 2024). Although considerable progress has been achieved in research pertaining to entophytic fungi, certain challenges and shortcomings persist. For instance, research has predominantly concentrated on specific plants or certain fungal types, and the stability and persistence of entophytic fungi in practical applications still necessitate ongoing research and exploration.

*Camellia luteflora* Li ex H.T. Chang, a rare and endemic plant in China, is a member of the Theaceae family, Camellia genus. It is currently confined to a narrow distribution along the banks of the Chishti River (Chen and Wang, 2016). Renowned for its golden flowers and broad leaves, *C. luteflora* possesses high ornamental value and holds promising prospects in landscaping, potting, and the fresh-cut flower industry (Zou, 2000). However, due to habitat fragmentation and its own biological characteristics (slow growth, susceptibility to pests and diseases (Liu et al., 2005), the wild population is diminutive, leading to its classification as Vulnerable (VU) by the International Union for Conservation of Nature (IUCN), and it is also a key plant for protection in Guizhou and Sichuan Province. Therefore, augmenting its growth rate and bolstering its resistance to pests and diseases are the primary strategies to ensure critical progress in the conservation of this species. In our preliminary work, Yi et al. (2024) have isolated and identified 35 strains of entophytic fungi of *C. luteflora* and discovered that 26 strains were inhibitory to at least one pathogenic fungus. Given the functional role of entophytic fungi in promoting plant growth and enhancing stress resistance, conducting an in-depth and systematic study of the growth-promoting characteristics of entophytic fungi in *C. luteflora* holds significant theoretical value and practical significance for developing scientific conservation strategies for this species.

This study aims to evaluate the plant-promoting characteristics of 35 entophytic fungi isolated from *C. luteflora*, including indole-3-acetic acid (IAA) production capacity, nitrogen fixation, protease activity, iron carrier production capacity, phosphorus solubilization, and potassium mobilization; Potential strains were formulated into microbial inocula, and their plant-promoting effects were validated through pot experiments; thereby screening the optimal strains for the development of novel entophytic fungal inocula. Through systematic research on the plant-promoting potential of entophytic fungi in *C. luteflora*, this study aims to provide a theoretical basis for the growth of *C. luteflora* and to promote the development of sustainable fungal inocula.

## 2 Materials and methods

### 2.1 Materials

#### 2.1.1 Fungal strains and plant material

The test subjects comprise 35 strains (Table 1) of entophytic fungi isolated from *C. luteflora*, preserved in the College of Life Sciences, Sichuan University, originating from our preliminary research conducted by Yi et al. (2024).

**Table 1:** Classification status of entophytic fungi strains of *C. luteflora.*

Serial number	Representative strain	Similar strain	Accession number	Homology (%)
1	XG-V-2	*Herpotrichiellaceae* sp. 2 KO-2013	AB847008.1	99
2	QG-IV-3	*Cadophora orchidicola*	MG062775.1	100
3	CG-II-1	*Helotiales* sp.	KX440158.1	99
4	DG-I-11	*Pezicula ericae*	MT626580.1	100
5	XY-V-3	*Ascomycota* sp. WP-2010	NR_184377.1	99
6	DJ-III-9	*Pestalotiopsis* sp.	MT102582.1	99
7	DJ-II-1	*Pestalotiopsis chamaeropis*	MH425902.1	99
8	DJ-IV-4	*Pestalotiopsis microspora*	KM041703.1	99
9	DJ-IV-10	*Pestalotiopsis oxyanthi*	KP900246.1	99
10	QJ-III-2	*Hypoxylon fragiforme*	KY703399.1	100
11	QY-II-4	*Annulohypoxylon stygium*	MW504640.1	99
12	DJ-I-4	*Xylaria arbuscula*	MZ853489.1	99
13	DY-IV-1	*Chaetomium subaffine*	MW008023.1	99
14	DY-II-1	*Nodulisporium* sp.	LC505317.1	99
15	DY-V-3	*Arthrinium arundinis*	MZ400522.1	99
16	DJ-I-2	*Fusarium graminearum*	MZ648312.1	100
17	XG-III-4	*Ilyonectria liriodendri*	MF445151.1	99
18	CG-IV-11	*Diaporthe hongkongensis*	MZ617453.1	99
19	CJ-II-2	*Diaporthe phaseolorum*	MT043783.1	99
20	DY-IV-12	*Diaporthe amygdali*	MK511798.1	99
21	DY-III-5	*Diaporthe biguttulata*	MT877049.1	99
22	CJ-IV-6	*Diaporthe discoidispora*	MH371253.1	99
23	QJ-I-8	*Phomopsis* sp.	MG832539.1	99
24	QJ-V-4	*Colletotrichum pseudomajus*	JX009424.1	99
25	QJ-IV-7	*Colletotrichum crassipes*	KY283783.1	99
26	CY-III-3	*Colletotrichum boninense*	MZ702124.1	100
27	CJ-I-8	*Colletotrichum acutatum*	MN429239.1	100
28	QY-IV-3	*Colletotrichum camelliae*	MK041380.1	100
29	CJ-V-1	*Colletotrichum siamense*	MW647834.1	99
30	XY-I-7	*Colletotrichum gloeosporioides*	KP900263.1	100
31	DJ-V-5	*Penicillium herquei*	OM278352.1	99
32	QY-II-6	*Aphanoascus verrucosus*	MW617041.1	99
33	XY-III-5	*Phyllosticta capitalensis*	MG954332.1	100
34	XY-II-2	*Cladosporium cladosporioides*	JX406506.1	100
35	XJ-II-1	*Acrocalymma medicaginis*	MW081367.1	100

The first two digits in the name of the representative strain indicate the acronym of the season and site, the middle Roman numeral indicates the number of the plant taken, and the last digit indicates the number of the tissue block to which it belongs.

Tomato seeds (*Solanum lycopersicum*. ‘Alisa Craig’) were provided by the School of Biological Sciences, Sichuan University (Chengdu, China).

#### 2.1.2 Soil substrates

Plant cultivation substrate: Peat, Vermiculite, Perlite, with a volume ratio of 3:1:1 (Jinyun County, Zhejiang Province, China; CPAI Flagship Store).

Solid Fermentation Substrate: Corn Cobs, Soybean Meal, Rice Hulls, Wheat Bran Powder, Soybean Powder, with a mass ratio of 10:4:2:2:2.

#### 2.1.3 Culture medium

All media were prepared with reagents from Chron Chemicals (Chengdu, China) unless stated otherwise:

Nitrogen fixation medium (Zhao and He, 2002): Sucrose (5.0 g), CaCO_3_ (5.0 g), CaSO_4_·2H_2_O (0.1 g), KH_2_PO_4_ (0.2 g), MgSO_4_·7H_2_O (0.2 g), NaCl (0.2 g), mannitol (10.0 g), agar (13.0 g), distilled water (1.0 L), pH 7.0–7.2;

Protease-producing medium (Yang et al., 2008): Skim milk powder (15.0 g; Solarbio, Beijing, China), agar powder (16.0 g), distilled water 1.0 L, pH 7.2;

Potassium solubilization medium (Han et al., 2011): Glucose (10.0 g), Na_2_HPO_4_ (0.2 g), MgSO_4_·7H_2_O (0.2 g), NaCl (0.2 g), CaSO_4_·2H_2_O (0.2 g), CaCO_3_ (5.0 g), agar (15.0 g), Potassium Feldspar (2.5 g, KAlSi₃O₈, Macklin, Shanghai, China), distilled water (1.0 L), pH 7.2;

Phosphorus dissolving medium (Shi et al., 2008): Glucose 10.0 g, (NH4)_2_SO_4_ (0.5 g), MgSO_4_·7H_2_O (0.3 g), MnSO_4_·4H_2_O (0.03 g), KCl (0.3 g), FeSO_4_·7H_2_O (0.03 g), NaCl (0.3 g), Ca _3_(PO_4_)_2_ (5.0 g), agar (20.0 g), distilled water (1.0 L), pH 7.0–7.5.

#### 2.1.4 Main reagents

All chemicals were purchased from Chron Chemicals (Chengdu, China) unless specified:

Reagents: Anhydrous Ethanol, Methanol, Tryptophan, 3.00% Sodium Hypochlorite Solution, Acetone, Ethyl Acetate, sodium hyposulfite Na_2_S_2_O_3_, 2,3,5-triphenyltetrazolium chloride (TTC) Solution (1 mg·mL^−1^), Phosphate Buffer Solution (PBS), Sulfuric Acid (1 mol·L^−1^), Succinic Acid (0.4 mol·L^−1^).

Salkowski colorimetric solution (Gordon and Weber, 1951): 500 mL of 35.00% sulfuric acid (H₂SO₄) was added to 10 mL of 0.5 mol·L^−1^ FeCl_3_ solution. This formulation enables stable detection of indole-3-acetic acid (IAA) at 530 nm.

CAS detection solution (Schwyn and Neilands, 1987): 83 μL of concentrated hydrochloric acid was added to 1.5 mL of 1 mmol-L-1 FeCl_3_, mixed well with 7.5 mL of 2 mmol·L^−1^ chromium azurite solution, 6.0 mL of 10 mmol·L^−1^ hexadecyltrimethylammonium bromide (HDTMA) solution, 4.307 g of anhydrous piperazine was dissolved in 30 mL of distilled water and then added to the mixture, and finally 6.25 mL of concentrated hydrochloric acid was added and the volume was fixed to 100 mL with distilled water.

### 2.2 Methods

#### 2.2.1 Evaluation of growth-promoting ability of entophytic fungi from *Camellia luteflora*

##### 2.2.1.1 Detection of IAA-producing activity by entophytic fungi from *Camellia luteflora*

Primary Screening: Using a 5 mm mycelial block as inoculum (1 block per vial), the endophytes were inoculated in 50 mL of potato dextrose liquid medium (PDW) (Hopebio, China) containing 0.5 g-L^−1^ tryptophan at 28 °C in the dark and shaken at 160 r min^−1^ for 7 days. The fermentation broth was filtered through a nonwoven cloth and centrifuged at 12,000 r·min^−1^ for 5 min. 1 mL of the supernatant was mixed with 2 mL of Salkowski colorimetric solution and incubated for 20 min in the dark. Potential IAA-producing fungi were screened by observing the red color development reaction.

Secondary Screening: Selected strains were cultured in triplicate under identical conditions, and the supernatant was collected according to method 2.3.1. One milliliter of supernatant was mixed with 1 mL of Salkowski’s reagent in equal volume, and the reaction was protected from light for 20 min, then the absorbance was measured at 530 nm using a UV–Vis spectrophotometer (Thermo, USA). The concentration of IAA was quantified by a standard curve (0–30 mg·L^−1^, *R*^2^ = 0.998), and a blank correction was performed throughout the experiment (Borah and Thakur, 2020).

##### 2.2.1.2 Assessment of nitrogen fixation capacity in entophytic fungi isolated from *Camellia luteflora*

All 35 fungal endophytes were inoculated under aseptic conditions with four 5 mm mycelial discs per plate (3 plates per strain) and incubated in the dark at 28 °C for 7 days. Subsequently, the fungal strains were sub-cultured onto fresh nitrogen-fixing agar plates, and this subculturing process was repeated for three consecutive generations (Dai et al., 2017). The ability of entophytic fungal strains to grow normally on nitrogen-free medium across all generations is indicative of nitrogen fixation capability.

##### 2.2.1.3 Evaluation of protease secretion activity in entophytic fungi isolated from *Camellia luteflora*

The 35 strains of entophytic fungi from *C. luteflora* were each inoculated onto protease activity agar plates within a laminar flow hood, with quadruplicate fungal discs per plate and triplicate plates per strain. Incubation was carried out in the dark at 28 °C for 7 days. The emergence of a transparent halo surrounding the fungal colonies signifies the secretion of proteases by the entophytic fungi. The diameter of the halo (d) and the diameter of the fungal colony (D) were measured, and the ratio (d/D) was calculated to evaluate the intensity of protease activity (Yang et al., 2008).

##### 2.2.1.4 Determination of siderophore production by entophytic fungi isolated from *Camellia luteflora*

The 35 strains of entophytic fungi from *C. luteflora* were inoculated into PDW broth, with triplicate cultures per strain, and incubated at 28 °C with agitation at 160 r·min^−1^ for 7 days. After filtration through a non-woven fabric, 5.0 mL of the supernatant was collected and centrifuged at 12,000 r·min^−1^ for 5 min. Thereafter, 3.0 mL of the supernatant was combined with 3.0 mL of CAS reagent. Following a 1-h incubation at room temperature in the dark, the absorbance (As) was measured at 630 nm using a UV–visible spectrophotometer, with distilled water as a blank for zero adjustment. In parallel, 3.0 mL of uninoculated PDW broth was mixed with 3.0 mL of CAS reagent, and the absorbance (Ar) was determined using the same protocol (Schwyn and Neilands, 1987). The siderophore activity of the entophytic fungi was calculated using the formula:

SU = [(Ar-As)/Ar] × 100%.

Where Ar represents the absorbance of the blank reagent and As represents the absorbance of the reagent mixed with the supernatant.

##### 2.2.1.5 Assessment of potassium solubilization potential in entophytic fungi isolated from *Camellia luteflora*

In a laminar flow hood, the 35 strains of entophytic fungi from *C. luteflora* were each inoculated onto potassium-solubilizing medium, with quadruplicate plugs per medium and triplicate sets per strain, and incubated at 28 °C in the dark for 7 days. Any lightening of the medium color or formation of transparent zones around the fungal plugs is indicative of potassium solubilization activity. The diameters of the transparent zones (d) and fungal plugs (D) were measured, and the (d/D) ratio was calculated to assess the strength of potassium solubilization (Bagyalakshmi et al., 2017).

##### 2.2.1.6 Assessment of phosphorus Solubilization potential in entophytic fungi isolated from *Camellia luteflora*

Similar to the potassium solubilization assessment, the phosphorus solubilization potential of each fungal strain was evaluated using the (d/D) ratio (Shi et al., 2008).

#### 2.2.2 Evaluation of the growth-promoting effects of entophytic fungi inocula from *Camellia luteflora* on potted tomato seedlings

##### 2.2.2.1 Fermentation process for the growth-promoting entophytic fungi inocula from *Camellia luteflora*

Strains CG-II-1, CJ-V-1, XY-II-2, DG-I-11, DY-II-1, and DJ-I-4, identified in section 2.2.1 as having growth-promoting potential, were inoculated into PDW medium and cultured at 28 °C with agitation at 160 rpm for 5 days. A total of 7 kg of solid fermentation substrate was weighed, sterilized at 121 °C under high pressure for 30 min, divided into seven equal portions on iron trays, mixed with deionized water at a 1:1 ratio, and supplemented with the fermentation liquid from the aforementioned six strains. The remaining substrate was treated with an equal volume of PDW medium as a control, mixed evenly, covered with two layers of gauze, and further cultured for 7 days in preparation for subsequent potting experiments.

##### 2.2.2.2 Tomato seedling cultivation

Fully plump and uniform tomato seeds were selected and subjected to surface sterilization using the following protocol: (1) immersion in 75% ethanol for 30 s, (2) treatment with 3% sodium hypochlorite solution for 10 min, and (3) thorough rinsing with sterile distilled water. The sterilized seeds were then dried on sterile absorbent paper. Sterilize the breeding substrate at 121 °C under high pressure for 30 min, allow to cool to room temperature, and fill into 50-hole trays (cell size: 48 mm diameter × 40 mm depth). Sow the sterilized tomato seeds in the 50-hole trays and incubate for germination. When the seedlings reach the three-leaf and one-heart stage, select seedlings with robust and uniform growth for the experimental treatments.

##### 2.2.2.3 Application of entophytic fungi inocula from *Camellia luteflora* for tomato seedling growth promotion

The experiment was conducted from August to October 2022 in the College of Life Sciences, Sichuan University. The potting experiment comprised the following treatments: CK, Helo., Csia., Ccla., Peri., Nodu., and Xarb., with 15 replicates for each. Each pot (10.7 cm diameter × 9.5 cm height) was planted with one tomato seedling. Each pot was filled with 0.5 kg of a culture substrate mixture (peat, vermiculite, perlite in a volume ratio of 3:1:1 (Wang, 2019) and 50 g of fermentation substrate, mixed uniformly (refer to Table 2 for details). The photoperiod was set to 12 h per day (9:00–21:00) with LED lights providing a photosynthetic photon flux density of 300 μmol·m^−2^·s^−1^ in a growth chamber. The environmental conditions were maintained at a daytime temperature of 24–26 °C, a nighttime temperature of 18–20 °C, a relative humidity of 65%, and the plants were watered every 2 days.

**Table 2:** Test treatments.

Treatment	Fermentation substrate
Helo.	CG-II-1 (*Helotiales* sp.)
Csia.	CJ-V-1 (*Colletotrichum siamense*)
Ccla.	XY-II-2 (*Cladosporium cladosporioides*)
Peri.	DG-I-11 (*Pezicula ericae*)
Nodu.	DY-II-1 (*Nodulisporium* sp.)
Xarb.	DJ-I-4 (*Xylaria arbuscula*)
CK	Blank fermentation substrate

Treatment groups were named by strain name abbreviations, and strain codes were replaced with strain abbreviations in the results for ease of reading.

##### 2.2.2.4 Assessment of tomato seedling growth parameters

On the 20th day after sowing, five uniformly growing seedlings were randomly selected from each treatment group, and plant height and stem thickness were measured every 5 days. On the 35th day after sowing, another five uniformly growing seedlings were selected from each treatment. To determine the dry mass, the samples were baked in an electric blast dryer at 75 °C until constant weight, and then the aboveground and belowground dry mass was determined using an electronic balance. Seedling vigor index was calculated as follows:

Seeding vigorous Index = (dry weight of underground parts/dry weight of aboveground parts + stem thickness/plant height) × total dry weight of the plant (Gong et al., 2019).

##### 2.2.2.5 Determination of tomato seedling gas exchange parameters

On the morning of the 36th day post-sowing, between 9:00 and 11:00, five uniformly grown seedlings from each treatment group were randomly selected. A portable photosynthesis system (Li-6800, LI-COR, USA) was used to measure the net photosynthetic rate (Pn), stomatal conductance (Gs), intercellular CO_2_ concentration (Ci), and transpiration rate (Tr) of the second leaf from the top. Measurements were conducted under a light intensity of 800 μmol·s^−1^, a flow rate of 500 μmol·s^−1^, a relative air humidity of 50%, a CO_2_ concentration of 400 μmol·mol^−1^, and a leaf chamber temperature of 25 °C. Each biological replicate was measured three times, and the mean values were calculated.

##### 2.2.2.6 Determination of tomato seedling chlorophyll content and root physiological activity

On the 36th day post-sowing, five uniformly grown seedlings per treatment were selected. The third healthy leaf from the top was sampled, and after the removal of the midrib, the chlorophyll a, chlorophyll b, and total chlorophyll content of the tomato leaves were determined using the acetone extraction method (Arnon, 1949). A section of the root with higher activity from the middle part was selected, and the root physiological activity was assessed using the triphenyl tetrazolium chloride (TTC) (Macklin, China) reduction method (Majdi, 1995).

#### 2.2.3 Data analysis

Raw data were summarized and counted by Excel (v. 2210 Build 16.0.15726.20188, Microsoft Office 365 MSO, USA). Statistical analyses of the data from each experiment were performed using an analysis of variance, and the mean comparison was performed using Duncan’s multiple range test (*p* < 0.05) in SPSS (v. 26.0.0, SPSS Inc., USA) software. Data values are presented as “mean ± standard deviation.” All images by origin2022 (v. 9.900225, OriginLab, USA).

## 3 Results

### 3.1 Assessment of growth-promoting abilities of entophytic fungi from *Camellia luteflora*

#### 3.1.1 Analysis of IAA production activity of entophytic fungi

**Figure 1:**
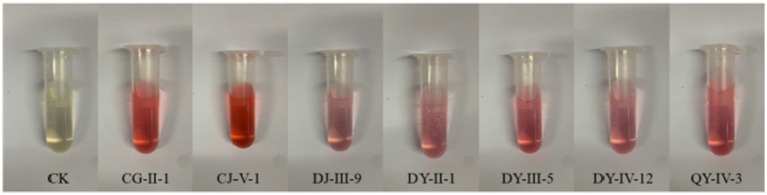
Preliminary screening results of IAA production activity of entophytic fungi from *C. luteflora.*

**Figure 2:**
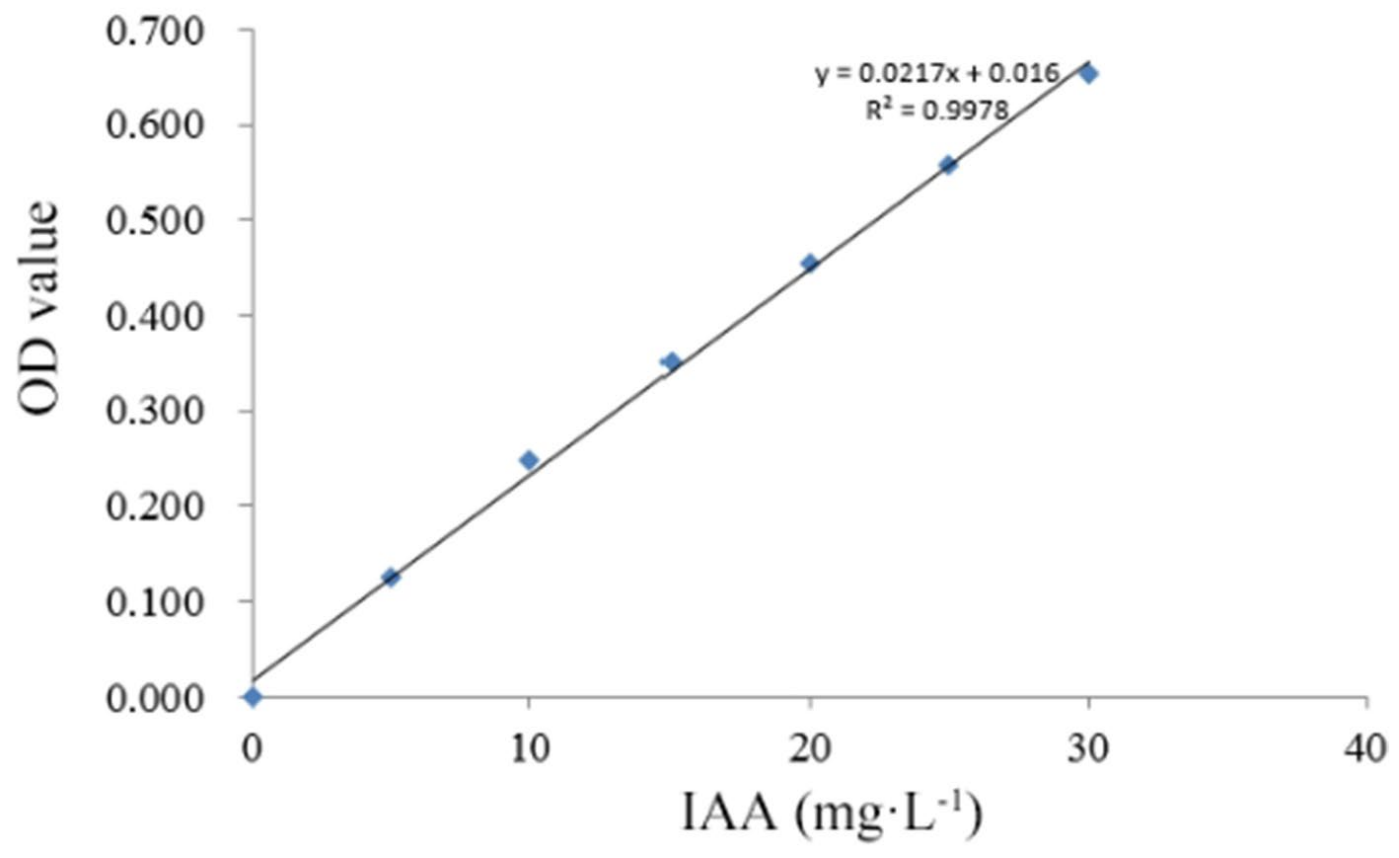
IAA standard curve diagram.

Among the 35 entophytic fungal strains isolated from *C. luteflora*, seven strains exhibited distinct colorimetric responses to the Salkowski reagent (Figure 1), indicating their potential for indole-3-acetic acid (IAA) biosynthesis. Quantitative analysis was performed using a UV spectrophotometer (OD₅₃₀) with a six-point calibration curve (5–30 mg·L^−1^ IAA standards). The standard curve demonstrated excellent linearity (*y* = 0.0217x + 0.016, *R*^2^ = 0.9978; Figure 2), enabling precise quantification of fungal IAA production.

As summarized in Table 3, the seven strains displayed significant variation in IAA yields, ranging from 14.44 to 51.60 mg·L^−1^. Notably, *C. siamense* (strain CJ-V-1) exhibited the highest production level (51.60 mg·L^−1^), which was statistically distinct from all other isolates (*p* < 0.05). It was followed by CG-II-1 (*Helotiales* sp.) with an IAA output of 33.83 mg·L^−1^, and then by QY-IV-3 (*C. camelliae*), DY-III-5 (*D. biguttulata*), DY-II-1 (*Nodulisporium* sp.), DY-IV-12 (*D. amygdali*), and DJ-III-9 (*Pestalotiopsis* sp.). These results highlight that *C. siamense* CJ-V-1 is a promising candidate strain for plant growth promotion.

**Table 3:** Evaluation results of the growth promoting ability of entophytic fungi from *C. luteflora.*

Strain no.	IAA concentration (mg·L^−1^)	Iron carrier SU value (%)	Nitrogen fixation capacity	Transparent ring diameter/strain diameter		
				Protease production	Potassium dissolving ability	Phosphorus solubilizing ability
CG-II-1	33.83 ± 0.74b	15.22 ± 0.13j	−	1.62 ± 0.03d	−	1.23 ± 0.02e
CG-IV-11	−	−	−	−	−	−
CY-III-3	−	−	−	1.15 ± 0.02gh	−	−
CJ-IV-6	−	44.93 ± 0.93c	+	1.04 ± 0.00i	−	−
CJ-II-2	−	40.88 ± 0.71d	+	−	−	−
CJ-V-1	**51.60 ± 1.60a**	−	+	1.12 ± 0.01ghi	−	−
CJ-I-8	−	−	+	1.34 ± 0.03e	−	−
XG-III-4	−	−	+	1.39 ± 0.04e	−	−
XG-V-2	−	13.30 ± 0.36 k	−	−	−	−
XY-III-5	−	−	+	−	−	−
XY-V-3	−	14.85 ± 1.17jk	+	−	−	−
XY-I-7	−	−	−	1.87 ± 0.11b	−	−
XY-II-2	−	48.68 ± 1.66b	+	1.91 ± 0.04b	−	−
XJ-II-1	−	−	+	1.74 ± 0.03c	−	−
QG-IV-3	−	17.25 ± 0.31i	+	−	−	−
QY-II-6	−	−	−	1.14 ± 0.01gh	−	−
QY-II-4	−	−	+	−	−	−
QY-IV-3	26.32 ± 0.36c	−	+	1.20 ± 0.02 fg	−	−
QJ-I-8	−	**52.89 ± 0.73a**	+	1.23 ± 0.00f	−	−
QJ-III-2	−	−	−	1.06 ± 0.00hi	−	−
QJ-V-4	−	28.71 ± 1.25f	+	1.12 ± 0.01ghi	−	1.76 ± 0.09b
QJ-IV-7	−	14.83 ± 0.38jk	+	1.07 ± 0.00hi	−	−
DG-I-11	−	31.24 ± 1.57e	+	**2.13 ± 0.05a**	1.21 ± 0.02c	1.46 ± 0.04c
DY-IV-1	−	−	+	1.37 ± 0.03e	−	1.10 ± 0.01f
DY-IV-12	16.97 ± 0.68de	−	+	1.09 ± 0.01hi	−	−
DY-V-3	−	−	+	1.08 ± 0.00hi	−	−
DY-II-1	18.51 ± 0.57d	−	+	1.07 ± 0.03hi	1.14 ± 0.03c	1.31 ± 0.04de
DY-III-5	24.19 ± 0.96c	17.46 ± 0.30i	+	1.06 ± 0.01hi	−	−
DJ-III-9	14.44 ± 1.35e	−	−	1.04 ± 0.00i	−	−
DJ-I-2	−	21.29 ± 0.25 h	+	1.11 ± 0.02hi	−	−
DJ-I-4	−	25.89 ± 1.05 g	+	1.13 ± 0.04ghi	**2.46 ± 0.19a**	**1.96 ± 0.07a**
DJ-II-1	−	−	−	1.14 ± 0.03gh	1.67 ± 0.10b	−
DJ-V-5	−	11.08 ± 0.45 L	−	−	−	1.35 ± 0.03d
DJ-IV-4	−	−	−	1.07 ± 0.00hi	1.34 ± 0.06c	−
DJ-IV-10	−	−	+	1.07 ± 0.01hi	−	−

Different lowercase letters indicate significant differences in different treatments (*p* < 0.05), “+” means positive, “−” means negative.

#### 3.1.2 Nitrogen fixation ability detection of entophytic fungi from *Camellia luteflora*

**Figure 3:**
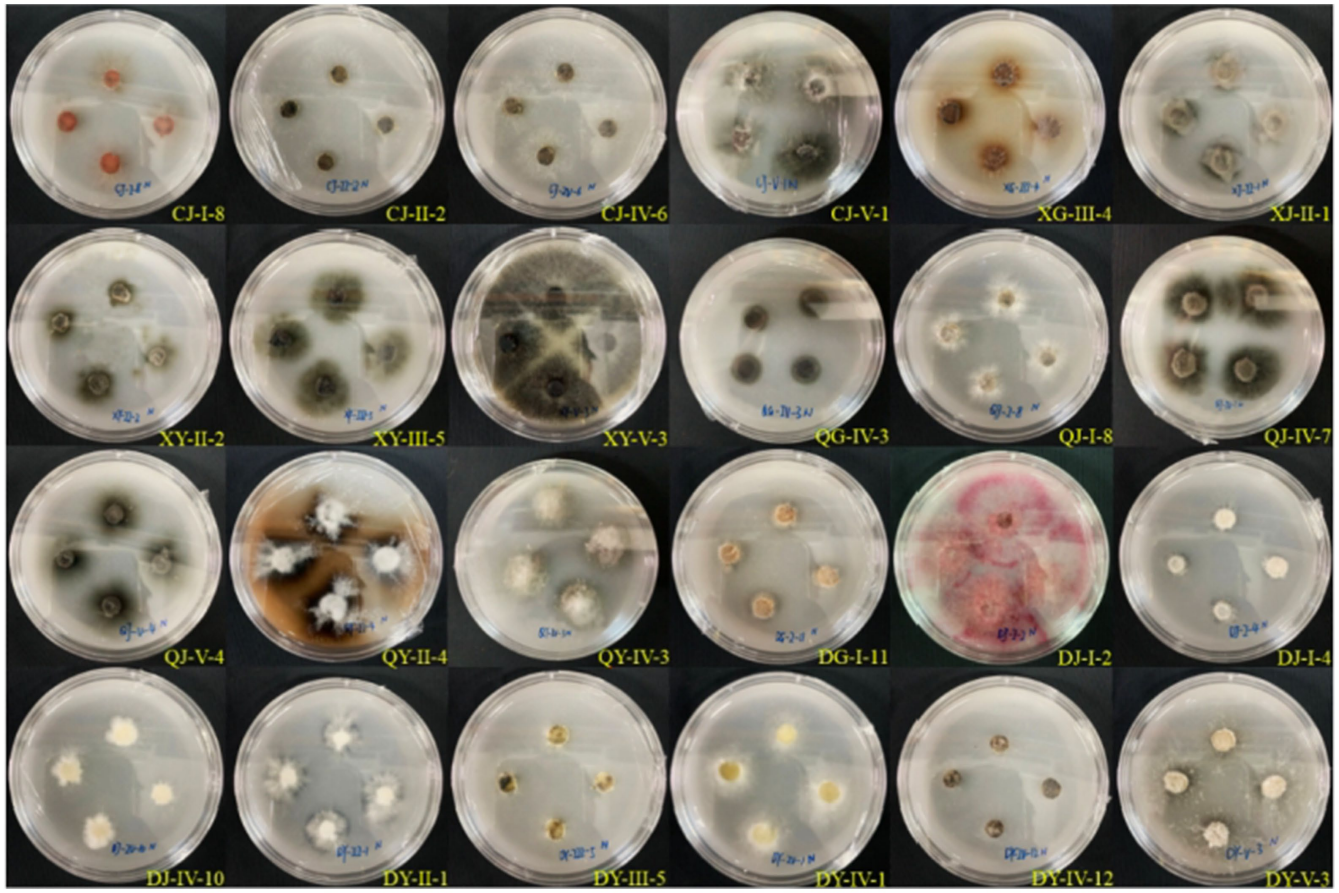
Test results of entophytic fungi nitrogen fixing ability of *C. luteflora.*

Thirty-five strains of entophytic fungi derived from *C. luteflora* were inoculated onto a nitrogen-fixing medium. Following three successive subcultures, 24 strains demonstrated the capacity to grow normally, as detailed in Table 3 and illustrated in Figure 3. This growth pattern suggests that these strains have the capability to fix nitrogen. Notably, strains DY-II-1, DJ-I-4, and DG-I-11 were observed to form transparent zones surrounding their colonies, a characteristic indicative of their additional ability to dissolve calcium carbonate (CaCO_3_) within the nitrogen-fixing medium.

#### 3.1.3 Assessment of protease activity in entophytic fungi isolated from *Camellia luteflora*

**Figure 4:**
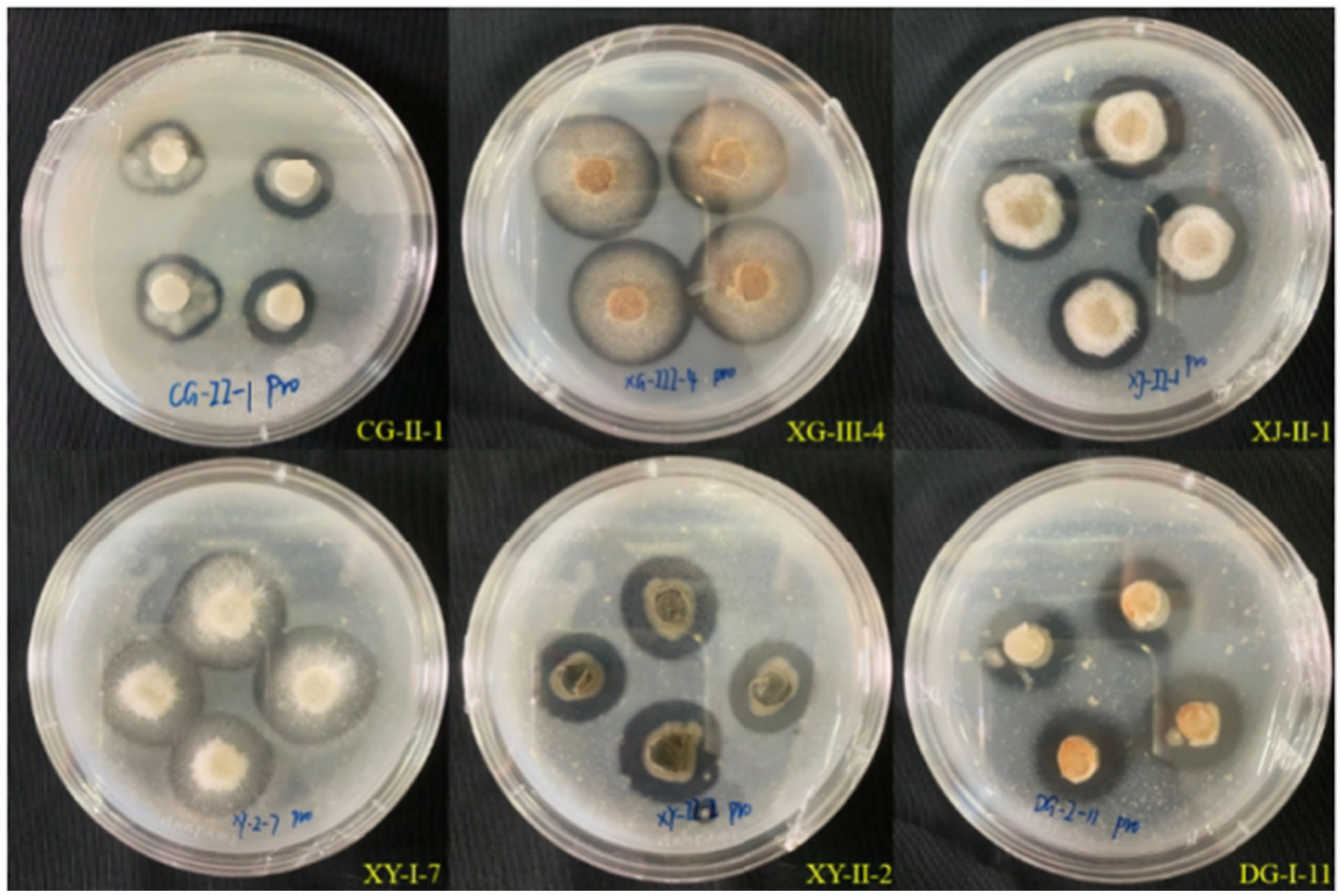
Results of the protease-producing ability of entophytic fungi in part of *C. luteflora.*

Upon inoculation of 35 strains of entophytic fungi from *C. luteflora* onto a medium designed to detect protease activity, the formation of transparent zones was observed encircling 27 strains, as depicted in Figure 4. This observation suggests that these strains have the capability to produce proteases and degrade proteins. Notably, strains DG-I-11, XY-II-2, and XY-I-7 demonstrated the most pronounced protease activity, with d/D ratios of 2.13, 1.91, and 1.87, respectively (Table 3).

#### 3.1.4 Assessment of Iron carrier production by entophytic fungi isolated from *Camellia luteflora*

Among the 35 entophytic fungal strains isolated from *C. luteflora*, 15 exhibited SU values exceeding 10.00%, signifying a robust capacity for iron carrier production. The SU values spanned from 11.08 to 52.89%, as detailed in Table 3. These strains, which include CG-II-1, CJ-IV-6, CJ-II-2, XG-V-2, XY-V-3, XY-II-2, QG-IV-3, QJ-I-8, QJ-V-4, QJ-IV-7, DG-I-11, DY-III-5, DJ-I-2, DJ-I-4, and DJ-V-5, demonstrated varying levels of iron carrier activity. Notably, strain QJ-I-8 had the highest SU value at 52.89%, closely followed by strain XY-II-2 with an SU value of 48.68%.

#### 3.1.5 Evaluation of potassium Solubilization capacity by Endophytic Fungi isolated from *Camellia luteflora*

**Figure 5:**
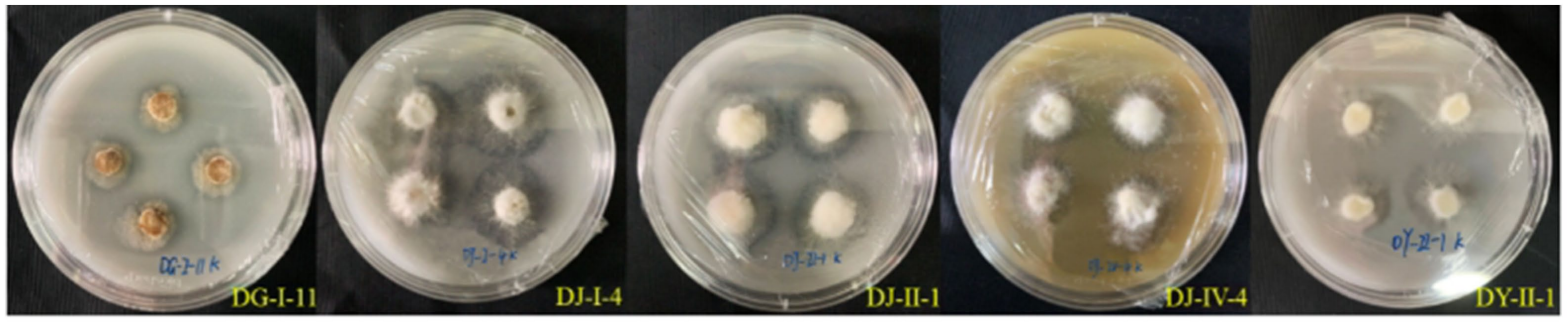
Potassium-solubilizing capacity of entophytic fungi of *C. luteflora.*

Five strains of entophytic fungi from *C. luteflora* displayed the formation of transparent zones on the potassium solubilization medium, as illustrated in Figure 5. These strains, identified as DG-I-11, DY-II-1, DJ-I-4, DJ-II-1, and DJ-IV-4, showed potential for potassium solubilization. Strain DJ-I-4 exhibited the most pronounced potassium solubilization ability, with the highest d/D value of 2.46, followed by strain DJ-II-1 with a d/D value of 1.67.

#### 3.1.6 Assessment of phosphate solubilization capacity in entophytic fungi isolated from *Camellia luteflora*

**Figure 6:**
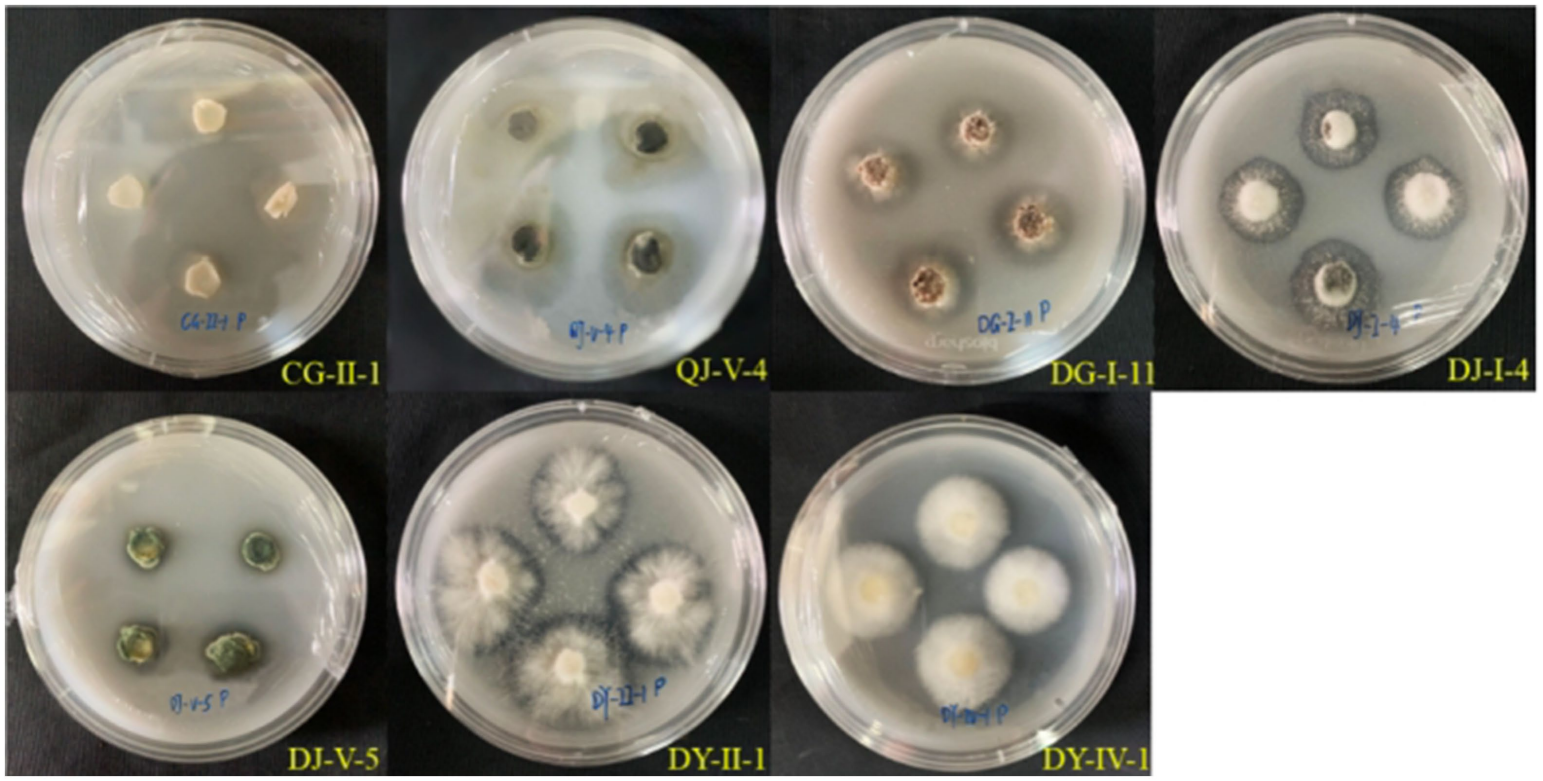
Phosphorus solubilizing capacity of the entophytic fungus *C. luteflora.*

During the phosphate solubilization assay, seven strains of entophytic fungi from *C. luteflora* manifested transparent zones surrounding their colonies on the medium, as depicted in Figure 4–6. These strains, which include CG-II-1, QJ-V-4, DG-I-11, DY-IV-1, DY-II-1, DJ-I-4, and DJ-V-5, indicate a capability for phosphate solubilization. Among these, strain DJ-I-4 demonstrated the most significant solubilization activity, with the highest d/D value of 1.96, followed by strain QJ-V-4 with a d/D value of 1.76.

### 3.2 Growth promotion effects of entophytic fungal inocula from *Camellia luteflora* on potted tomato seedlings

#### 3.2.1 Impact of entophytic fungal inocula on the growth parameters of potted tomato seedlings

**Figure 7:**
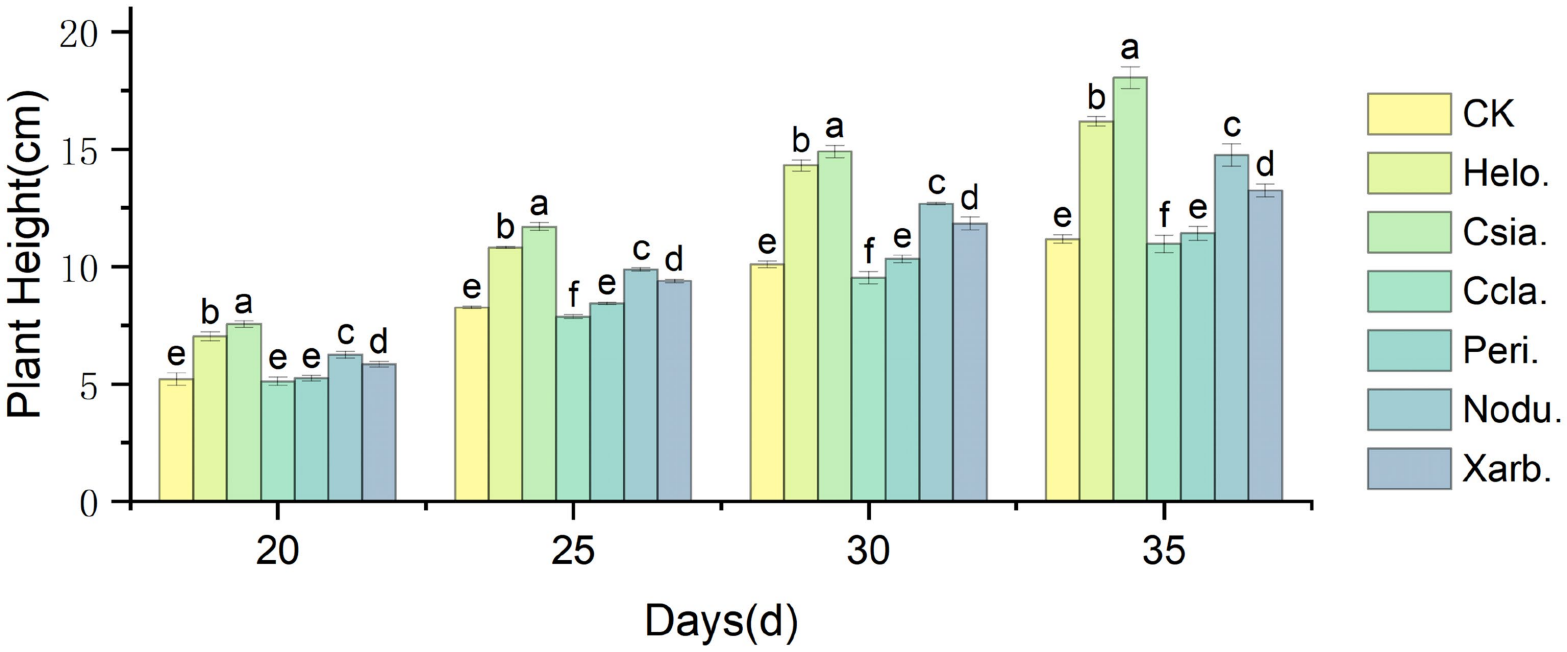
Variation trend of tomato seedling height in different treatment groups. Different lowercase letters indicate significant differences in different treatments (*p* < 0.05).

**Figure 8:**
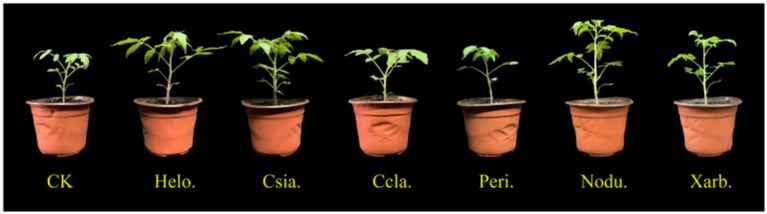
Growth of tomato seedlings in each treatment group on the 35th day after sowing.

As illustrated in Figure 7, the height of tomato seedlings across various treatment groups increased over time, with significant divergences emerging as the plants developed. At the seedling stage (35 days post-sowing), the height of the Csia. tomato seedlings (18.05 cm) surpassed that of the control (CK, 11.18 cm) by 61.44%, and the Csia. seedlings remained significantly taller than other treatments and the control from days 20–35 (*p* < 0.05). The heights of Helo. (16.19 cm), Nodu. (14.75 cm), and Xarb. (13.25 cm) seedlings also increased by 44.80%, 31.93%, and 18.52%, respectively, over CK. The height of Peri. tomato seedlings (11.43 cm) was not significantly different from CK, whereas Ccla. seedlings (10.97 cm) showed a 1.87% reduction compared to CK (Figure 8).

**Figure 9:**
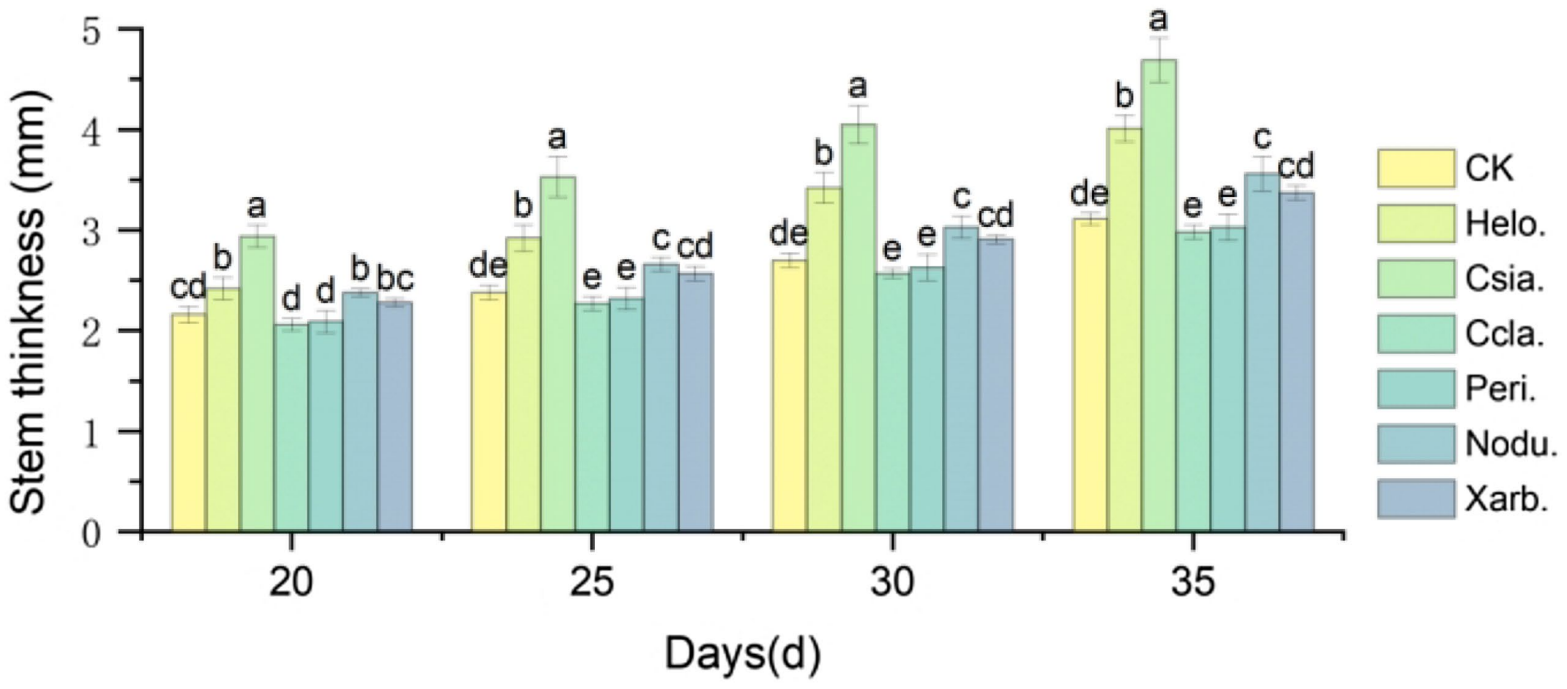
Variation trend of stem diameter of tomato seedlings in different treatment groups. Different lowercase letters indicate significant differences in different treatments (*p* < 0.05).

Figure 9 shows that as the growth period progressed, the stem thickness of tomato seedlings in the different treatment groups increased, with more pronounced differences among the groups. Between days 20–35, the stem thickness of Csia. tomato seedlings was significantly greater than that of other treatments and the control (*p* < 0.05). Except for day 20, Helo. tomato seedlings had a significantly greater stem thickness than Ccla., Peri., Nodu., Xarb., and CK on all other dates (*p* < 0.05). The stem thickness of Ccla., Peri., and Xarb. tomato seedlings did not significantly differ from CK during the 20 to 35 days post-sowing period. At 35 days post-sowing, the stem thickness of Helo., Csia., Nodu., and Xarb. tomato seedlings increased by 28.94% (4.01 mm), 50.80% (4.69 mm), 14.47% (3.56 mm), and 8.34% (3.37 mm), respectively, compared to CK (3.11 mm) (refer to Figures 4–8).

As shown in Table 4, aboveground and belowground dry mass of Helo., Csia., Nodu. and Xarb. tomato seedlings were significantly higher (*p* < 0.05) than that of CK, whereas the dry mass of Ccla. and Peri. tomato seedlings did not differ significantly from that of CK. Aboveground and belowground dry mass of Csia. tomato seedlings were increased by 373.86% and 262.5%, respectively, compared to CK. Among all the treatments, Csia. tomato seedlings had the highest dry mass, which was significantly better than other treatments and CK (*p* < 0.05), followed by Helo. whole plant dry mass of Helo., Nodu. and Xarb. tomato seedlings was increased by 296.88, 186.46 and 194.79%, respectively, compared to CK.

**Table 4:** Dry and fresh weight of tomato seedlings in different treatment groups.

Treatment	Above-ground dry mass/g	Subterranean dry mass/g	Whole plant dry mass/g
CK	0.088 ± 0.002de	0.008 ± 0.000d	0.096 ± 0.002de
Helo.	0.351 ± 0.005b	**0.030 ± 0.002a**	0.381 ± 0.002b
Csia.	**0.417 ± 0.007a**	**0.029 ± 0.001a**	**0.446 ± 0.006a**
Ccla.	0.092 ± 0.003d	0.009 ± 0.001d	0.101 ± 0.003d
Peri.	0.081 ± 0.004e	0.007 ± 0.000d	0.088 ± 0.004e
Nodu.	0.256 ± 0.007c	0.019 ± 0.001c	0.275 ± 0.007c
Xarb.	0.260 ± 0.005c	0.023 ± 0.002b	0.283 ± 0.007c

Different lowercase letters indicate significant differences in different treatments (*p* < 0.05).

#### 3.2.2 Impact of various entophytic fungal inocula on chlorophyll content in potted tomato seedlings

**Figure 10:**
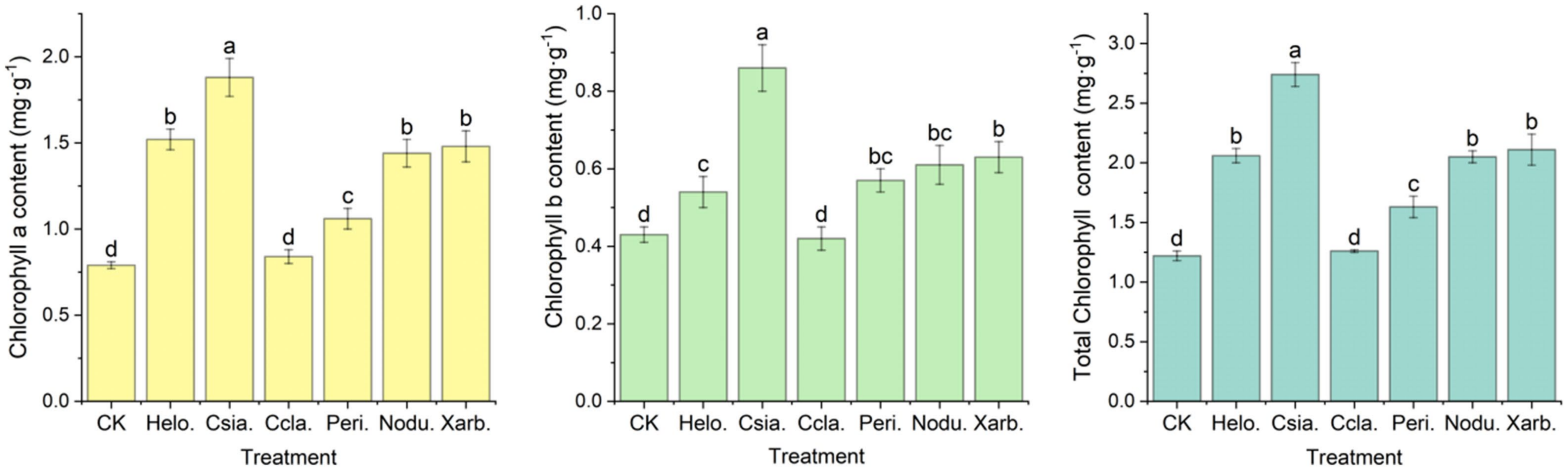
Chlorophyll content of tomato seedlings in different treatment groups. Different lowercase letters denote significant differences among various treatments (*p* < 0.05), with chlorophyll a, chlorophyll b, and total chlorophyll content presented from left to right.

As evidenced in Figure 10, with the exception of the Ccla. treatment, all other treatment groups exhibited significantly elevated levels of chlorophyll a, chlorophyll b, and total chlorophyll compared to the control group (CK). The Csia. treatment group demonstrated the highest chlorophyll a content at 1.88 mg·g^−1^, which was significantly greater than that of other treatments and the CK (*p* < 0.05). The chlorophyll a content in the Helo., Nodu., and Xarb. groups, averaging 1.52, 1.44, and 1.48 mg·g^−1^ respectively, did not differ significantly from each other but was markedly higher than the Ccla., Peri., and CK groups (*p* < 0.05), with Ccla. and Peri. registering the lowest at 0.84 and 1.06 mg·g^−1^ respectively, and CK at 0.79 mg·g^−1^. The Peri. group showed a significant increase compared to Ccla. and CK (*p* < 0.05), although no significant difference was observed between Ccla. and CK. Similarly, the Csia. group’s chlorophyll b content at 0.86 mg·g^−1^ was significantly higher than all other treatments and the CK (*p* < 0.05). The Helo., Peri., Nodu., and Xarb. groups had chlorophyll b levels of 0.54, 0.57, 0.61, and 0.63 mg·g^−1^ respectively, which were not significantly different from one another but were significantly higher than the Ccla. and CK groups (*p* < 0.05), with Ccla. at 0.42 mg·g^−1^ and CK at 0.43 mg·g^−1^. The total chlorophyll content followed a similar pattern of variation to that of chlorophyll a.

#### 3.2.3 Impact of diverse entophytic fungal inocula on gas exchange parameters of potted tomato seedlings

Data presented in Table 5 indicate that the utilization of various entophytic fungal entophytic fungal inocula has significantly affected the photosynthetic characteristics of tomato seedlings. Specifically, treatments Helo. and Csia. have been associated with the most pronounced enhancement in net photosynthetic rate, stomatal conductance, intercellular CO_2_ concentration, and transpiration rate, surpassing the outcomes of other treatments and the control group (CK) at a statistically significant level (*p* < 0.05). Treatment Nodu. has demonstrated a significant elevation in net photosynthetic rate, stomatal conductance, and transpiration rate when compared to treatments Ccla., Peri., Xarb., and CK (*p* < 0.05). Additionally, Nodu. has shown a markedly higher intercellular CO_2_ concentration compared to Ccla., Peri., and CK (*p* < 0.05). For treatment Xarb., there was a significant increase in intercellular CO_2_ concentration and transpiration rate relative to Ccla., Peri., and CK (*p* < 0.05). Treatment Peri., aside from displaying a net photosynthetic rate significantly lower than that of CK (*p* < 0.05), did not exhibit significant differences from Ccla. and CK in the remaining parameters under consideration.

**Table 5:** Gas exchange parameters of tomato seedlings in different treatment groups.

Treatment	Net photosynthetic rate (NPR)/μmol CO_2_·m^−2^·s^−1^	Stomatal conductance/mol H_2_O·m^−2^·s^−1^	Intercellular CO_2_ concentration/μmol CO_2_·mol^−1^	Rate of transpiration/mmol H_2_O·m^−2^·s^−1^	Water use efficiency (WUE)/mmol mol^−1^
CK	17.20 ± 0.23c	0.21 ± 0.00d	235.68 ± 4.03e	6.11 ± 0.16e	**2.82 ± 0.03a**
Helo.	**20.66 ± 0.50a**	0.31 ± 0.01b	**317.56 ± 4.28a**	8.09 ± 0.32b	2.56 ± 0.06de
Csia.	**21.32 ± 0.70a**	**0.35 ± 0.01a**	304.61 ± 4.44b	**8.57 ± 0.18a**	2.49 ± 0.02e
Ccla.	16.84 ± 0.30 cd	0.23 ± 0.00d	227.30 ± 7.23e	5.94 ± 0.14e	**2.84 ± 0.04a**
Peri.	16.31 ± 0.33d	0.22 ± 0.01d	231.46 ± 3.57e	6.05 ± 0.08e	2.70 ± 0.01b
Nodu.	18.79 ± 0.39b	0.27 ± 0.01c	255.97 ± 1.94d	7.24 ± 0.27c	2.60 ± 0.05 cd
Xarb.	17.46 ± 0.26c	0.23 ± 0.00d	267.34 ± 6.27c	6.59 ± 0.06d	2.65 ± 0.02bc

Different lowercase letters indicate significant differences in different treatments (*p* < 0.05).

#### 3.2.4 Influence of assorted entophytic fungal inocula on the seedling vigor index and root physiological activity of potted tomato seedlings

**Figure 11:**
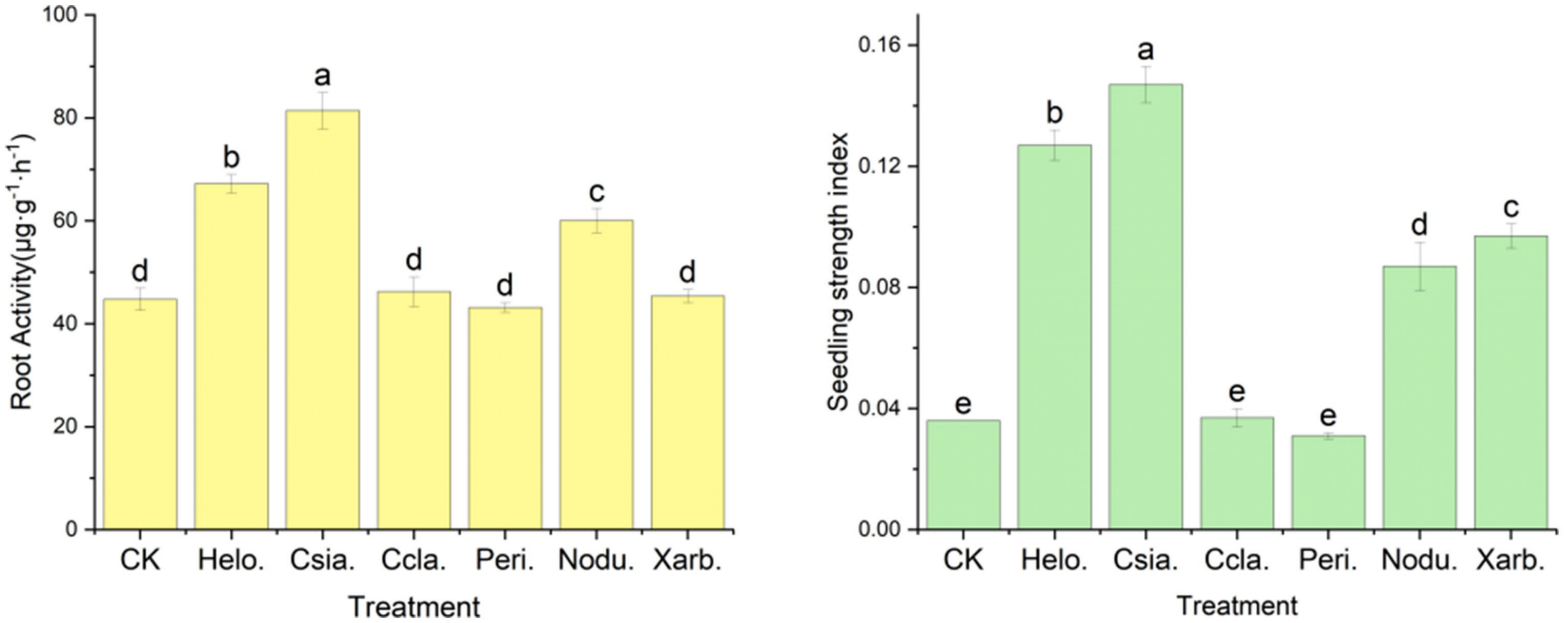
Effects of different entophytic fungi biologic fertilizer on seedling strength index and root physiological activity of potted tomato seedlings. Different lowercase letters indicate significant differences in different treatments (*p* < 0.05).

Figure 11 illustrates that the Csia. treatment group exhibited the most robust root physiological activity, with a measurement of 81.43 μg·g^−1^·h^−1^, which was significantly superior (*p* < 0.05) to all other treatments and the control group (CK), which recorded a value of 44.78 μg·g^−1^·h^−1^. The Helo. and Nodu. groups followed with root physiological activity of 67.23 μg·g^−1^·h^−1^ and 60.04 μg·g^−1^·h^−1^, respectively. In contrast, the Ccla., Peri., and Xarb. groups displayed root physiological activity of 46.24 μg·g^−1^·h^−1^, 43.17 μg·g^−1^·h^−1^, and 45.38 μg·g^−1^·h^−1^, respectively, which were not significantly different from the control.

The seedling vigor index, a crucial metric for assessing the growth of tomato seedlings, revealed the following order of magnitude from highest to lowest: Csia. (0.147) > Helo. (0.127) > Xarb. (0.097) > Nodu. (0.087) > Ccla. (0.037) > CK (0.036) > Peri. (0.031). Here, the Csia., Helo., Xarb., and Nodu. groups demonstrated significantly higher indices compared to the control (*p* < 0.05), while the Ccla. and Peri. groups were not significantly distinct from the control.

## 4 Discussion

Endophytic fungi are widely colonized in various plant tissues and possess the important ability to synthesize plant growth-regulating substances, particularly indole-3-acetic acid (IAA), gibberellin (GA), and cytokinin (CTK). These substances effectively promote physiological processes such as cell elongation, division, and differentiation, thereby accelerating plant growth and development (Waqas et al., 2012). Auxins, particularly IAA, act as ubiquitous plant hormones that modulate a plethora of physiological responses through signal transduction pathways, significantly influencing plant cell growth and division (Johnson and Crosby, 1963; Spaepen et al., 2007). However, the ability to produce IAA is confined to a subset of entophytic fungi isolated from plants, with notable examples from the genera *Aspergillus, Alternaria, Colletotrichum, Fusarium, Mortierella,* and *Penicillium*. For instance, *Fusarium tricinctum* RSF-4 L and *Alternaria alternata* RSF-6 L isolated from *Solanum nigrum* exhibited IAA production capacities of 54 mg·L^−1^ and 30 mg·L^−1^, respectively, with their culture filtrates significantly promoting rice seedling growth (Khan et al., 2015). Optimizing the fermentation conditions for growth-promoting bacteria has been demonstrated to enhance mycelial growth, IAA biosynthesis, and overall growth enhancement. *Ceratobasidium* sp. PJ3 isolated from the stems of ‘Hongxia’ *Anoectochilus roxburghii* achieved an IAA yield of 106.7 μg·mL^−1^ through optimized cultivation conditions (Chen et al., 2022). Similarly, *Colletotrichum fructicola* CMU-A109 exhibited a significant increase in IAA production from 662.96 ± 56.18 μg/mL to 1205.58 ± 151.89 μg·mL^−1^ after 26 days of cultivation at 30 °C in a medium containing L-tryptophan (8 mg·mL^−1^) (Numponsak et al., 2018).

These findings highlight the importance of optimizing the cultivation conditions for the high IAA-yielding strains CJ-V-1 and CG-II-1 in future studies. Since mycelial dry weight was not systematically measured in this study, IAA yield is reported as concentration per unit culture volume (mg·L^−1^) rather than biomass yield (mg·L^−1^). Future studies will integrate biomass weight quantification methods to enable rigorous comparative analysis of biosynthetic efficiency across different studies.

The role of entophytic fungi in promoting plant growth is not limited to the secretion of IAA, but also includes the direct promotion of plant growth through the secretion of organic acids, enzymes, and other substances. These substances can break down insoluble nutrients in the soil, improve the utilization of phosphorus and potassium, fix nitrogen, promote the conversion of iron ions, and thus promote plant growth and enhance its stress resistance.

Proteases play a crucial role in soil nutrient cycling by catalyzing the decomposition of organic matter into available nutrients, thereby enhancing soil fertility. A plethora of studies have identified various fungal species, including *Penicillium bilaiae, Talaromyces flavus, Mortierella hyalina, Paecilomyces variabilis*, and others, as potent protease producers. Notably, *Penicillium* and *Aspergillus* have demonstrated remarkable protease production capabilities (Bezerra et al., 2021). Our study revealed that 27 strains possessed protease production capabilities, with DG-I-11 exhibiting the highest enzyme activity, followed by XY-II-2 and XY-I-7.

Although nitrogen-fixing bacteria dominate the biological nitrogen fixation process, recent studies have shown that certain entophytic fungi, particularly arbuscular mycorrhizal fungi (AM fungi), also possess nitrogen-fixing capabilities. A notable example is the entophytic fungus *Phomopsis liquidambaris*, which has been shown to increase peanut (*Arachis hypogaea* L.) yields and significantly promote nodulation and nitrogen fixation, independent of nitrogen fertilizer application (Xie et al., 2019). Our study identified 24 strains with nitrogen-fixing capabilities, capable of converting inorganic nitrogen to organic nitrogen.

Plant biotrophic fungi can enhance the uptake of mineral nutrients by promoting nutrient transport and activating inter-root nutrients (Chen et al., 2023). But Phosphorus-solubilizing fungi constitute a minor fraction of the total soil fungal population, with entophytic fungi being significant contributors. Key entophytic Phosphorus-solubilizing fungi belong to the genera *Penicillium, Aspergillus, Piriformospora, Curvular*ia, and the class of arbuscular mycorrhizal (AM) fungi. A notable example is the *Xylaria schweinitzii* strain YS-2-2, isolated from the roots of *Phoebe bournei*, which demonstrates significant growth-promoting effects through enhanced phosphorus solubilization (Yao, 2020). It has been shown that various Trichoderma fungi can solubilize different phosphates, enhancing phosphorus uptake by plants and thus promoting plant growth (Bononi et al., 2020). Similarly, although potassium is geochemically abundant in soils, it mainly exists in the form of stable aluminosilicate minerals, limiting its plant availability. Fungal-mediated mineral weathering and organic acid secretion have been shown to effectively convert fixed potassium into plant-available forms. Zhan et al. (2017) identified two strains, *Aspergillus awamori* MQ013 and *Aspergillus niger* MQ039, with phosphorus-solubilizing, potassium-solubilizing, and IAA-secreting activities, which significantly promoted the growth of maize seedlings. In our study, a total of 7 phosphorus-solubilizing strains and 5 potassium-solubilizing strains were identified, among which the DJ-I-4 strain exhibited the most prominent phosphorus and potassium solubilization capacity among all strains. Notably, DJ-I-4 exhibited the highest phosphorus and potassium solubilizing capacity among all strains. The entophytic fungus *Xylaria arbuscula*, isolated from *Cupressus lusitanica*, has been reported to produce bioactive substances such as cytochalasin (Amarala et al., 2014), making DJ-I-4 a promising candidate for further development and utilization.

Iron is crucial for chlorophyll synthesis and enhancing plant stress tolerance, but its bioavailability is limited in alkaline or high-phosphorus soils, leading to plant iron deficiency. Fungal-produced iron carriers play a significant role in plant iron transport (Huong et al., 2022). To date, 500 diverse siderophores have been documented, with a majority being of entophytic origin, found in various microbial sources, including mycorrhiza and orchidaceous fungi (Neilands, 1981). The highest siderophore percentages were reported in *Penicillium chrysogenum* (CAL1) at 57%, *Aspergillus sydowii* (CAR12) at 49%, and *Aspergillus terreus* (CAR14) at 46% by CAS liquid assay (Chowdappa et al., 2020). In this study, a total of 15 strains exhibited iron carrier production activity, with iron carrier utilization (SU) values ranging from 11.08% to 52.89%. Given that iron carrier-producing microorganisms can also enhance plant disease resistance (Deng et al., 2023), further study of the disease resistance characteristics of these strains is of great significance.

Our research identified six entophytic fungal strains (*Helotiales* sp. CG-II-1, *Colletotrichum siamense* CJ-V-1, *Cladosporium cladosporioides* XY-II-2, *Pezicula ericae.* DG-I-11, *Nodulisporium* sp. DY-II-1, and *Xylaria arbuscula* DJ-I-4) with significant plant growth-promoting potential. When these strains were formulated into entophytic fungal inocula and applied to tomato seedlings, they exhibited varying degrees of growth-promoting effects, with Colletotrichum siamense CJ-V-1 (Csia.) showing the most significant effects across multiple physiological parameters. The Csia. treatment resulted in remarkable improvements in plant growth traits, including a 61.45% increase in height, 50.80% increase in stem diameter, and 373.86 and 262.5% increases in above- and below-ground biomass, respectively, compared to controls. These growth enhancements were accompanied by significant improvements in photosynthetic performance, with Csia. and Helotiales sp. CG-II-1 (Helo.) showing 23.95% and 20.11% increases in net photosynthetic rate (Pn), respectively. The superior performance of these treatments appears closely linked to their indole-3-acetic acid (IAA) production capacity, as evidenced by our assays showing CJ-V-1 and CG-II-1 as the most potent IAA producers among the tested strains.

Under the treatment conditions of this study, the factors directly affecting net photosynthetic rate include: fungal fertilizer type, chlorophyll content, stomatal conductance and transpiration rate, and intercellular carbon dioxide concentration. Several studies have demonstrated that the application of exogenous indole-3-acetic acid (IAA) can significantly enhance the net photosynthetic rate of plants by modulating endogenous hormones, enhancing photosynthesis, and other mechanisms. For instance, in the study of *Syringa villosa*, IAA treatment resulted in significantly higher levels of endogenous IAA and gibberellic acid (GA3) than the control, while abscisic acid (ABA) levels were significantly lower; it increased net photosynthetic rate (Pn), and also increased stomatal conductance (Gs) and transpiration rate (Tr) (Jin et al., 2023). In *Brassica juncea*, it was shown that IAA improves photosynthesis efficiency by increasing chlorophyll content, enhancing the activity of key enzymes carbonic anhydrase (CA) and nitrate reductase (NR), and carboxylation efficiency (CE) (Ahmad et al., 2001). Additionally, plant growth regulators can affect the net photosynthetic rate through various mechanisms such as regulating stomatal conductance, enhancing photosynthetic parameters, improving photosynthetic efficiency, and increasing photosynthetic pigment content (Müller and Munné-Bosch, 2021).

Therefore, we concluded that IAA secreted by entophytic fungi also enhances the photosynthetic efficiency of plants by increasing chlorophyll content, net photosynthetic rate (Pn), stomatal conductance (Gs), and transpiration rate (Tr), and intercellular CO_2_ concentration. Harman et al. suggest that certain specific *Trichoderma* strains are capable of up-regulating photosynthesis-related genes and pigments in plants through symbiosis with the root system, thereby enhancing photosynthetic efficiency (Harman et al., 2021). Although IAA-mediated signaling may initiate enhanced photosynthesis, improved nutrient acquisition through fungal-assisted mineralization may synergize with photosynthesis efficiency through indirect pathways (e.g., root development for mineral uptake). Thus, it is imperative to explore the molecular mechanisms by which CJ-V-1 and CG-II-1 improve photosynthetic efficiency.

Water use efficiency (WUE) is a key parameter for assessing the water use efficiency of plants, reflecting the efficiency of water use and the strength of drought tolerance. In general, species with high water use efficiency tend to be more resilient; gas exchange parameters have a significant effect on WUE of plants. While fungal treatments, particularly Helo. and Csia., significantly enhanced photosynthetic performance, this improvement came at the cost of reduced WUE due to concomitant increases in stomatal conductance and transpiration rates. This trade-off aligns with established C3 plant physiology principles, where maximal WUE typically occurs at gs < 0.4 mol m^−2^ s^−1^ (Hetherington and Woodward, 2003), and mirrors observations in endophyte-colonized tall fescue (Elbersen and Charles, 1996). The underlying mechanisms likely involve fungal-mediated alterations in stomatal regulation, potentially through changes in hormonal signaling or hydraulic properties. Interestingly, these results contrast with reports of improved WUE in other endophyte-plant systems (Rodriguez et al., 2008; Zheng et al., 2021), suggesting that the net effect on water relations depends on specific fungal-plant combinations and environmental conditions. Furthermore, it has been posited that both over-fertilization and waterlogging can result in a decrease in WUE (Zhang et al., 2020). Therefore, it is essential to establish various concentration gradients of fungal fertilizers to thoroughly investigate the relationship between gas exchange parameters and WUE. By doing so, we can identify the optimal concentrations of Helo. and Csia. that can enhance both photosynthetic efficiency and water-use efficiency, thereby providing a theoretical foundation for their practical application.

From Table 3, it is evident that CJ-V-1 and CG-II-1 exhibited the highest IAA-producing capabilities, while other growth-promoting attributes were either absent or not pronounced. Consequently, we posit the Helo. and Csia. play a pivotal role in promoting plant growth through IAA. This is consistent with previous reports: inoculation with the entophytic fungus *Alternaria* LQ1230, which produces IAA, can simultaneously enhance wheat growth capacity and drought tolerance (Qiang et al., 2019). It is worth noting that studies have found that entophytic fungi can also improve tomato performance through various mechanisms, including promoting tomato growth under salt stress conditions (Gaber et al., 2023) and maintaining plant growth in contaminated soil (Berthelot et al., 2017). Therefore, it is imperative to explore the potential application and underlying mechanisms of Helo. and Csia. in plant stress tolerance, particularly in the context of drought resistance.

*Colletotrichum siamense* is a phytopathogenic fungus that is prevalent in many tropical and subtropical regions globally and is known to cause anthracnose diseases in a diverse range of plants. It is found worldwide and can infect a wide range of plants, causing leaf disease, such as *Persea americana* (Li et al., 2022), *Salix matsudana* (Zheng et al., 2021), and *Etlingera elatior* (Duarte et al., 2022). Current research is predominantly focused on species diversity, infection mechanisms, and control strategies (Wang et al., 2023). Helotiales, a relatively understudied fungal group, has been recognized in recent research for its significant potential in enhancing host plant nutrition (Bruyant et al., 2024). It is noteworthy that DY-II-1 (*Nodulisporium* sp.), not only produces IAA but also fixes nitrogen, solubilizes potassium, phosphorus, and produces protease. Its ability to promote the growth of fungi fertilizers is second only to that of Helo. and Csia., and it also exhibits a certain level of antibacterial activity against strawberry anthracnose (Yi et al., 2024). Thus, it represents an entophytic fungus that warrants further research, development, and utilization.

In this study, we report for the first time that CJ-V-1 (*Colletotrichum siamense*) and CG-II-1 *(Helotiales* sp.), isolated from the spring stems and roots of *C. luteflora*, are capable of producing IAA and possess a strong capacity to promote plant growth. Comprehensive studies on these strains will contribute to elucidating the molecular mechanisms and metabolic pathways involved in IAA production, offering novel insights and perspectives for fundamental research in the fields of plant hormone biology and microbial metabolic engineering.

## 5 Conclusion

Among the 35 strains of entophytic fungi isolated from *C. luteflora*, seven exhibited IAA-producing activity. Strain CJ-V-1 demonstrated the highest IAA production at 51.60 mg L^−1^, which was significantly distinct from the other strains (*p* < 0.05), followed by CG-II-1 with an IAA production of 33.83 mg L^−1^. Twenty-four strains were capable of nitrogen fixation, 27 strains exhibited protease production capabilities, and the strain DG-I-11 displayed the most robust protease-producing ability, with a d/D ratio of 2.13. Fifteen strains with siderophore utilization (SU) values above 10.00% showed high iron carrier production ability, with SU values ranging from 11.08% to 52.89%. Strain QJ-I-8 had the most potent iron carrier production ability, with an SU value of 52.89%. Five strains had the ability to solubilize potassium, and seven strains could solubilize phosphorus, which were capable of dissolving potassium feldspar and Ca (PO)_4_, respectively. There were three strains that had the concurrent ability to solubilize phosphorus and solubilize potassium, with strain DJ-I-4 being the most potent, exhibiting d/D values of 1.96 and 2.46 for solubilizing phosphorus and potassium, respectively. The entophytic fungal inocula produced by the entophytic fungi strains CG-II-1, CJ-V-1, and DY-II-1 through fermentation significantly enhanced the chlorophyll content in tomato seedlings, increased the net photosynthetic rate, and promoted the development of their root systems and the accumulation of biomass. The order of their growth-promoting effects was CJ-V-1 > CG-II-1 > DY-II-1.

## Data Availability

The original contributions presented in the study are included in the article/supplementary material, further inquiries can be directed to the corresponding author.
